# Curcumin Regulates Cancer Progression: Focus on ncRNAs and Molecular Signaling Pathways

**DOI:** 10.3389/fonc.2021.660712

**Published:** 2021-04-12

**Authors:** Haijun Wang, Ke Zhang, Jia Liu, Jie Yang, Yidan Tian, Chen Yang, Yushan Li, Minglong Shao, Wei Su, Na Song

**Affiliations:** ^1^ Department of Pathology, Key Laboratory of Clinical Molecular Pathology, The First Affiliated Hospital of Xinxiang Medical University, Xinxiang, China; ^2^ School of Basic Medical Sciences, Xinxiang Medical University, Xinxiang, China; ^3^ Department of Mental Health, The Second Affiliated Hospital of Xinxiang Medical University, Xinxiang, China; ^4^ Institute of Precision Medicine, Xinxiang Medical University, Xinxiang, China

**Keywords:** curcumin, cancer, ncRNA, miRNA, signaling pathway, review

## Abstract

Curcumin [(1E,6E) ‑1,7‑bis(4‑hydroxy‑3‑methoxyphenyl) hepta‑1,6‑diene‑3,5‑ dione] is a natural polyphenol derived from the rhizome of the turmeric plant *Curcuma longa*. Accumulated evidences have presented curcumin’s function in terms of anti-inflammatory, antioxidant properties, and especially anti-tumor activities. Studies demonstrated that curcumin could exert anti-tumor activity *via* multiple biological signaling pathways, such as PI3K/Akt, JAK/STAT, MAPK, Wnt/β-catenin, p53, NF-ĸB and apoptosis related signaling pathways. Moreover, Curcumin can inhibit tumor proliferation, angiogenesis, epithelial-mesenchymal transition (EMT), invasion and metastasis by regulating tumor related non-coding RNA (ncRNA) expression. In this review, we summarized the roles of curcumin in regulating signaling pathways and ncRNAs in different kinds of cancers. We also discussed the regulatory effect of curcumin through inhibiting carcinogenic miRNA and up regulating tumor suppressive miRNA. Furthermore, we aim to illustrate the cross regulatory relationship between ncRNA and signaling pathways, further to get a better understanding of the anti-tumor mechanism of curcumin, thus lay a theoretical foundation for the clinical application of curcumin in the future.

## Introduction

Malignant tumor is a tremendous threat to human health with high heterogeneity. Most cancer retain untreatable although the understanding of cancer on a molecular level has been improved in the past years. According to the latest data released by World Health Organization (WHO), the number of deaths due to malignant tumors is still increasing every year (1). In exploring the way to treat cancer, scientists have been constantly finding and developing new drugs to treat cancer, including chemotherapy, radiotherapy, targeted drugs, and immunotherapy, and so on. Accumulated studies have shown that some natural compounds have a good effect on anti-tumor therapy, which provides us an alternative way to treat and manage cancer.

Curcumin[(1E,6E)‑1,7‑bis(4‑hydroxy‑3‑methoxyphenyl)hepta‑1,6‑diene‑3,5‑dione], an active component extracted from the rhizomes of *Curcuma longa* Linn (2), presents some favorable results in terms of anti-proliferation, anti-inflammation, anti-angiogenesis and anti-oxidation (3–8). Curcumin represents a promising phytochemicals in the field of anticancer to be used alone or combined with other drugs, which can affects multiple signaling pathways and molecular targets involved in cancer pathogenesis including NF-kB, MAPK, PTEN, p53, and ncRNA network (9).

A non-coding RNA (ncRNA) is a functional RNA molecule that is transcribed from DNA but not translated into proteins, which include microRNA (miRNA), small interfering RNA (siRNA), PIWI-interacting RNAs (piRNAs) and long noncoding RNAs (lncRNAs) (10–13). More and more studies have demonstrated that ncRNA plays an epigenetic regulatory role in the anti-cancer effect of curcumin, involving with histone modification, DNA methyltransferases, and regulation of microRNA (miRNA) expressions (14–18).

Although there have been reviews and talk about the role of curcumin in anti-tumor, it is mainly through explaining the regulation of curcumin in anti-tumor on related signal pathways, such as proliferation, invasion/migration, autophagy, apoptosis and other pathways (19–23), but there is no the relationship between ncRNA and pathway was clarified. In this review, we provide insights into the vital role and the signal pathways of curcumin in anti-cancer, and focus on the molecular mechanism of curcumin *via* ncRNA in regulating tumor development.

## PI3K-Akt Signal Pathway

The PI3K/AKT pathway is one of the major intracellular signaling pathways which has diverse downstream effects on basic intracellular functions, including cell proliferation, growth, apoptosis, epithelial-mesenchymal transition(ETM), metabolism and motility (24–26). The aberrant activation of the signaling pathway is often closely related to the occurrence and development of human cancers (27).

Studies show that miRNA-203,206 and miRNA-192-5p are very important tumor-inhibiting ncNRA. In a variety of tumors, they can inhibit tumor proliferation, invasion, migration, EMT, angiogenesis, *etc* (28–34). Curcumin could inhibit the cell proliferation and induce apoptosis of non-small cell lung cancer (NSCLC) cells *via* up-regulating the expression of miRNA-206/miRNA-192-5p and suppressing the PI3K/AKT/mTOR signaling pathway (35, 36). On the contrary, miR-206/miRNA-192-5p inhibitors could reverse the above results, following with the mTOR and Akt phosphorylation levels increase, which could promote cancer cell migration and invasion. Furthermore, miR-206/miRNA-192-5p mimics can also enhance curcumin inhibitory effect (37, 38). Curcumin might be a very effective agent for the treatment of NSCLC.

The most common genetic alteration activating PI3K is the genetic ablation of the tumor suppressor phosphatase and tensin homolog (PTEN), a negative regulator of PI3K-AKT signaling (39). miR-21 is one of the most frequently observed abnormal miRNAs in human cancers and is one of the first miRNAs described as oncogenic ncRNA (40–44). A large-scale miRNA analysis of 540 samples from six different types of solid tumors showed that miR-21 is the only up-regulated miRNA in all cancer types (45).

Curcumin can effectively repress the miR-21/PTEN/Akt molecular pathway to inhibit cell proliferation and induce apoptosis in gastric cancer cells. The p-Akt protein expression is negatively correlated with curcumin dose, with curcumin levels increase, PTEN expression increased and miR-21 levels decreased (46–48). Qiang et al.’s study showed that curcumin can play a synergistic role with other chemotherapy drugs. Curcumin could facilitate the apoptosis effect of PD98059, a potent and selective inhibitor of mitogen-activated protein kinase, *via* the miR-21/PTEN/Akt pathway (48). Diphenyl difluoroketone (EF24), curcumin analogue, function to enhance apoptosis by inhibiting miR-21 in DU145 human prostate cancer cells and B16 mouse melanoma cells (49).

Curcumin inhibits the proliferation of bladder cancer cells (50). Curcumin mediated the expression of miR-203 increase by inducing hypomethylation of miR-203 promoter. The target genes of miR-203, Akt2, Src, Jun, and Survivin, were repressed in mRNA and protein level, leading to proliferation decrease and apoptosis increase of bladder cancer cells.

Curcumin significantly enhanced the apoptosis of renal carcinoma Caki cells induced by dual PI3K/Akt and mTOR inhibitor NVP-BEZ235. Curcumin suppress Bcl-2 level and the stability of Mcl-1 protein with p53 dependent mode. It is noteworthy that NVP-BEZ235 alone has no effect on the death of human renal cell carcinoma Caki cells, but NVP-BEZ235 combined with curcumin contributed cell apoptosis significantly with Bcl-2 and Mcl-1 reduction (51).

## JAK-STAT Signal Pathway

Constitutive activation of the Janus kinase/signal transducers and activators of transcription (JAK/STAT) pathway was first recognized as being related with malignancy in the 1990’s (52). JAK-STAT pathways can be activated and employed by diverse cytokines, growth factors, and related molecules, that in turn phosphorylates their targets STATs (53, 54). Activated STATs, as the form of dimerization, are translocated into the nucleus and initiated the gene transcription.

The effects of JAK/STAT signaling on tumor cell survival, proliferation and invasion have made the JAK/STAT pathway a hotspot target for tumor targeted therapy and drug development (55, 56). The expression of miR-99a has been studied in many human cancers. It has been reported to be down-regulated in several types of cancer, including non-small cell lung cancer (NSCLC), liver cancer, bladder cancer, *etc (* 57–59). These findings indicate that miR-99a is widely down-regulated in human cancers, indicating the potential role of miR-99a as a tumor suppressor.

Curcumin can inhibit the proliferation, migration, invasion and promote apoptosis of retinoblastoma cells, which function through up-regulating the miR-99a expression and then inhibiting JAK/STAT signaling pathway (60). In addition, curcumin can suppress the cell proliferation by down-regulations of cyclinD1 and up-regulations of p21 expression. In one of studies showed that curcumin could reduce the anti-apoptotic protein Bcl-2, enhance the pro-apoptotic protein Bax and cleaved-caspase-3/9 expression level, ultimately promoted the apoptosis of retinoblastoma cells. Furthermore, curcumin can synergistically enhance the anti-tumor activity of cisplatin on papillary thyroid cancer (PTC) cells and tumor stem cell like cells by inhibiting the activity of STAT3, suggesting that curcumin combined with chemotherapy drugs may play a better therapeutic effect *via* JAK/STAT signaling pathway (61).

## Wnt/β-Catenin Signal Pathway

Wnt/β-catenin signaling pathway, also called the canonical Wnt signaling pathway, is a highly conserved pathway in evolution. Activation of Wnt pathways can modulate cell proliferation, apoptosis, differentiation, migration, invasion, genetic stability, cell renewal. β-catenin is the core component of the cadherin complex and its stabilization is essential for the activation of Wnt/β-catenin signaling. T-cell factor/lymph enhancer factor-1 (TCF/LEF1) is a transcription complex that mediates the transcription of genes triggered by classic Wnt pathway. When the Wnt pathway is activated, β-catenin can bind to the TCF/LEF1 complex by translocating into the nucleus and bind to the target gene DNA to initiate the transcription of related genes, such as: cyclinD1, c-Myc, MMPs (62–66). Wnt/β-catenin plays a vital role in tumor occurrence and treatment response.

The anti-cancer effect of curcumin is released by inhibiting the Wnt/β-catenin pathway. In osteosarcoma, curcumin could promote the protein level of reversion-inducing cysteine-rich protein with kazal motifs (RECK) by reducing the expression of miR-21, further inhibited the Wnt pathway related proteins, such as: β-catenin, GSK-3β (67). Curcumin suppresses the NSCLC cell proliferation, migration, invasion and viability in a dose‑dependent manner (68). Mechanistic analysis indicated that curcumin enhanced the expression level of miR−192−5p and decreased the expression of c−Myc. In addition, gene knockout miR‑192‑5p can rescue the curcumin-induced decrease of β-catenin, cyclin D1 and c‑Myc expression levels. These findings indicate that the up-regulation of miR‑192‑5p, induced by curcumin, inhibits NSCLC cells with inactivation of Wnt/β‑catenin signaling pathway and down-regulation of c-Myc transcription. Dou et al. documented that curcumin suppresses colon cancer by inhibiting Wnt/β-catenin pathway *via* down-regulating miR-130a, which may serve as a new target for colon cancer treatment (69).

As known, the lncRNA series (such as HOXA-AS2, HOTAIR and DANCR) function as oncogenes to promote cancer development and metastasis (70–72). Curcumin induced cell cycle arrest and apoptosis in hepatocellular carcinoma (HCC) by down-regulating lincROR which has been demonstrated to activate Wnt/β-catenin signaling (73). Shao et al. documented that curcumin may suppress cell proliferation of HCC cell line SMMC-7721, Huh-7, LO2 in a dose-dependent manner, the anti-proliferation effect of curcumin was partly caused by S phase arrest. The apoptosis rate of SMMC-7721 and Huh-7 cells with curcumin treatment was significantly higher than that in DMSO treatment group. Moreover, curcumin inhibits cell viability through lincROR/β-catenin regulatory pattern., Interestingly, the overexpression of lincROR partially reversed the inhibition of cell proliferation and rescue the inactivation of Wnt/β-catenin in curcumin-treated HCC cells (74). Liu et al. also found that curcumin suppressed the proliferation and tumorigenicity through a ceRNA effect of miR-145 and lncRNA-ROR in prostate cancer (75).

The cancer cells lose the characteristics of epithelial cells and acquire the mesenchymal phenotype, these changes are conducive to tumor cell invasion of surrounding tissues. The pathways associated with the EMT process have multiple signal cascades including the Wnt/β-catenin pathway. The curcumin analog EF24 shows effective anti-tumor activity by preventing the cell cycle and inducing cell apoptosis in melanoma (76). EF24 could inactivate the Wnt pathway *via* up-regulating the expression of miR-33b, which was able to inhibit EMT in melanoma cell lines through downregulation of the mesenchymal markers, Vimentin and N-cadherin. In addition, EF24 could decrease the phosphorylation of STAT3 and inhibit the HMGA2 expression, an oncological transcription factor, which promotes the proliferation of cancer cells and affect the progress of cell cycle in ovarian cancer cells and leukemia cells (77–79). In tamoxifen-resistant breast cancer cells, Curcumin attenuates lncRNA H19-induced epithelial-mesenchymal transition by the increase of N-cadherin and the decrease of E-cadherin (80).

## MAPK Signal Pathway

The mitogen-activated protein kinase (MAPK) cascade is a key signaling pathway that regulates various cellular processes (such as proliferation, differentiation, transformation, apoptosis and stress response) under normal and pathological conditions (81–84). Curcumin can inhibit proliferation and migration of human glioblastoma cells (85). miR-378 is considered to be a tumor suppressor miRNA, which has the ability to inhibit tumor cell proliferation, invasion and migration (86, 87). Li et al., proved that miR-378 is a downstream miRNA of glioblastoma multiforme, and it can enhance the inhibitory effect of curcumin on the growth of glioblastoma. Its inhibitory effect was enhanced in the stable U87 cells expressing miR-378 *in vitro* and *in vivo* compared with control cells. Moreover, it was found that curcumin can inhibit the expression of phosphorylated p38 at the protein level in U87 cells. miR-378 can counteract p38 inhibitory effect by increasing the phosphorylation of p38, further enhancing the sensitivity of cells to curcumin (85). Yu et al., showed that curcumin reduced the retinoblastoma cell viability and induced the apoptosis of Y79 cells through the activation of JNK and p38 MAPK pathways (88). Curcumin inhibits the growth and invasion of human monocytic leukemia SHI-1 cells *in vivo* by regulating MAPK and MMP signal transduction. The administration of curcumin significantly inhibited tumor growth, which showed that the tumor weight significantly decreased from 0.67 g (control) to 0.47 g (15 mg/kg) and 0.35 g (30 mg/kg). Curcumin inhibits the expression of PCNA in tumor tissues, and increases the degree of TUNEL and the staining of cleaved caspase-3. Curcumin treatment can be involved in the downregulation of NF-κB and ERK signals, and simultaneously activate p38 and JNK. And curcumin would attenuate the expression levels of MMP2, MMP9 and vimentin to affect the EMT process (89). Curcumin has also been shown to inhibit the growth of human A549 lung adenocarcinoma cells and induce apoptosis with a dose-dependent manner. Treatment of A549 cells by curcumin causes the increase of cytosolic cytochrome c and loss of mitochondrial membrane potential. Curcumin-induced apoptosis was accompanied by alterations in intracellular oxidative stress-related enzymes, including decreased reactive oxygen species (ROS), malondialdehyde and 4-hydroxynonenal levels, increased superoxide dismutase (SOD), accompanied with the alteration of MAPK signaling pathway factors c-JNK, p38, ERK and apoptosis related protein, such as cleaved-caspase-3,9, Bcl-2, Bax, HSP70, and poly (ADP-ribose) polymerase (PARP) (90, 91). It may be owing to the biological activities of curcumin metabolites, which have cytotoxic effects represented by overhaul mutated DNA and remove free radicals, respectively (92, 93). Another study of NSCLC showed that curcumin strengthened the chemosensitivity of cisplatin the in A549 and H1703 cells *via* the downregulation of XRCC1. Breast cancer study revealed that the MAPK pathway mediated the down-regulation of EZH2, contributing to the anti-proliferative effects of curcumin against breast cancer (94). Taken together, curcumin exerts its anti-cancer role *via* MAPK signaling pathway in multiple types of cancer.

## p53 Signal Pathway

TP53 (p53), also described as the ‘guardian of the genome’, is the single most frequently altered gene in kinds of human cancers, the p53 mutations were observed in approximately 50% of all invasive tumors (95–98). p53 is a tumor suppressor gene involved in a variety of cellular mechanisms, including DNA repair, apoptosis, and cell cycle arrest. p53 mutations in breast cancer have been associated with lower survival rates and resistance to conventional therapies. Curcumin interferes with the proliferation of breast cancer by up-regulating pro-apoptotic proteins (such as p53 and Bax) and down-regulating anti-apoptotic proteins (such as MDM2 and Bcl-2). Studies showed that curcumin inhibited the proliferation of estrogen-receptor-positive MCF-7 human breast cancer cells from bisphenol A (BPA)-induced *via* modulating oncogenic miR-19 (99). Li et al., demonstrated that BPA exhibited estrogenic activity by increasing the proliferation of MCF-7 cells and triggering the cell transition from G1 to S phase. Curcumin inhibited the proliferation of BPA on MCF-7 cells. Meanwhile, BPA-induced upregulation of oncogenic miR-19a can be reversed by curcumin, including dysregulation of miR-19-related downstream proteins, such as PTEN, p-Akt, p-MDM2, p53, and proliferative nuclear antigen. These results indicate that curcumin can inhibit the proliferation of breast cancer mediated by BPA by regulating the miR-19/PTEN/AKT/p53 pathway (99).

It has been reported that curcumin leads to a markedly inhibition of cell growth, and promotes apoptosis in non-small cell lung cancer cells *via* p53-miR-192-5p/215-XIAP pathway (100). X-linked inhibitor of apoptosis (XIAP) is a member of the inhibitor of apoptosis family of proteins (IAP) and plays an important role in the process of resisting apoptosis (101, 102). More importantly, curcumin-induced increase in miR-192-5p/215 level was closely related to intracellular p53 status, only in wild-type cells (A549), while, curcumin does not cause an increase of miR-192-5p/215 in cells without p53 expression (H1299, p53-null). With miRBase, TargetScan, PicTar and miRDB target databases and luciferase assay, it was further discovered that XIAP is the direct target gene of miR-192-5p/215. Curcumin promotes cell apoptosis by increasing the level of miR-192-5p/215, which binds directly to resists apoptosis XIAP mRNA (100). Xu et al., demonstrated that curcumin mediates the sensitivity of bladder cancer cells to radiotherapy by activating p53. The combination of 10 µM curcumin and radiation was more effective in reducing miR-1246 expression, cell viability and colony formation than curcumin or radiation alone. Inhibition of miR-1246 significantly reduces the cell viability and colony formation of T24 and HT-1376 cells. Luciferase reporter assay showed that miR-1246 can directly inhibit the expression of p53 gene. Finally, miR-1246 participates in the anti-cancer effects of curcumin and radiation by targeting to inhibiting the translation of p53 gene in bladder cancer cells (103).

Furthermore, mechanism studies have shown that curcumin can inhibit the growth of gastric cancer cells by down-regulating lncRNA H19 which was proved to be a oncomir (104, 105). Gao et al. proved that curcumin inhibits the proliferation of SGC7901 gastric cancer cells by repressing the expression of lncRNA H19 and enhancing the expression of p53. Meantime, curcumin can enhance the expression ratio of Bax/Bcl-2, both of them are downstream molecules of p53, thereby promoting cell apoptosis. Additionally, curcumin can also reduce the expression of oncogene c-Myc which indicate that curcumin inhibits the proliferation of gastric cancer cells by down-regulating the c-Myc/lncRNA H19 pathway (106).

## NF-ĸB Signal Pathway

NF-κB (NF-κB) is a transcription factor involved in a wide variety of biological activity. Growing evidence support its pivotal role in many steps of tumorigenesis and chemoresistance. Aberrant or constitutive NF-kB activation has been detected in many human malignancies (107–109). Suppression of the NF-kB signaling pathway has turned out to be a potential therapeutic approach for cancer treatment.

Studies showed that curcumin inhibits invasion and proliferation of cervical cancer cells *via* impairment of NF-kB and Wnt/β-catenin pathways (110). Curcumin effectively inhibits oncogenic NF-kB signaling and restrains stemness features in liver cancer (111). Under hypoxic conditions, curcumin would attenuate the malignancy of pancreatic cancer cell invasion and EMT by interfering with tumor−stromal crosstalk *via* the ERK/NF−κB axis. Li et al., demonstrated that curcumin inhibits the activation and migration of pancreatic stellate cells (PSC), which play an important role in pancreatic cancer progression. Moreover, curcumin inhibits pancreatic cancer cell invasion, EMT and alters the expression of E-cadherin, vimentin, and MMP-9. Furthermore, curcumin can counteract the increased levels of p-ERK and p-NF-κB (112). Xiang et al., documented that curcumin also can repress the proliferation, migration and apoptosis by regulating the NF‑κB signaling pathway in human colorectal carcinoma HCT−116 cells (113). In T47D breast cancer cells, curcumin treatment for 48 h, prevented human autocrine growth hormone (GH) signaling mediated NF-kB activation and miR-183-96-182 cluster stimulated epithelial mesenchymal transition (114). Animal study showed that curcumin suppressed the paclitaxel-induced NF-kB in breast cancer cells and strengthened the growth inhibitory effect of paclitaxel in a breast cancer nude mice mode. Compared with either drug alone, the combined treatment of paclitaxel and curcumin can significantly suppress cell proliferation and reduce tumor size, increase cell apoptosis, and reduce the expression of matrix metalloproteinase 9 (MMP9) (115). Studies demonstrated that curcumin inhibits both cyclo-oxygenase-2 enzyme (COX-2) and NF-kB. Therefore, it decreases binding of NF-kB to DNA and overpasses chemoresistance phenomenon in cancer cells (92, 93, 116, 117).The targeting of the NF-ĸB signaling by curcumin might be a treatment option for cancer.

## Summary and Prospect

**Figure 1:**
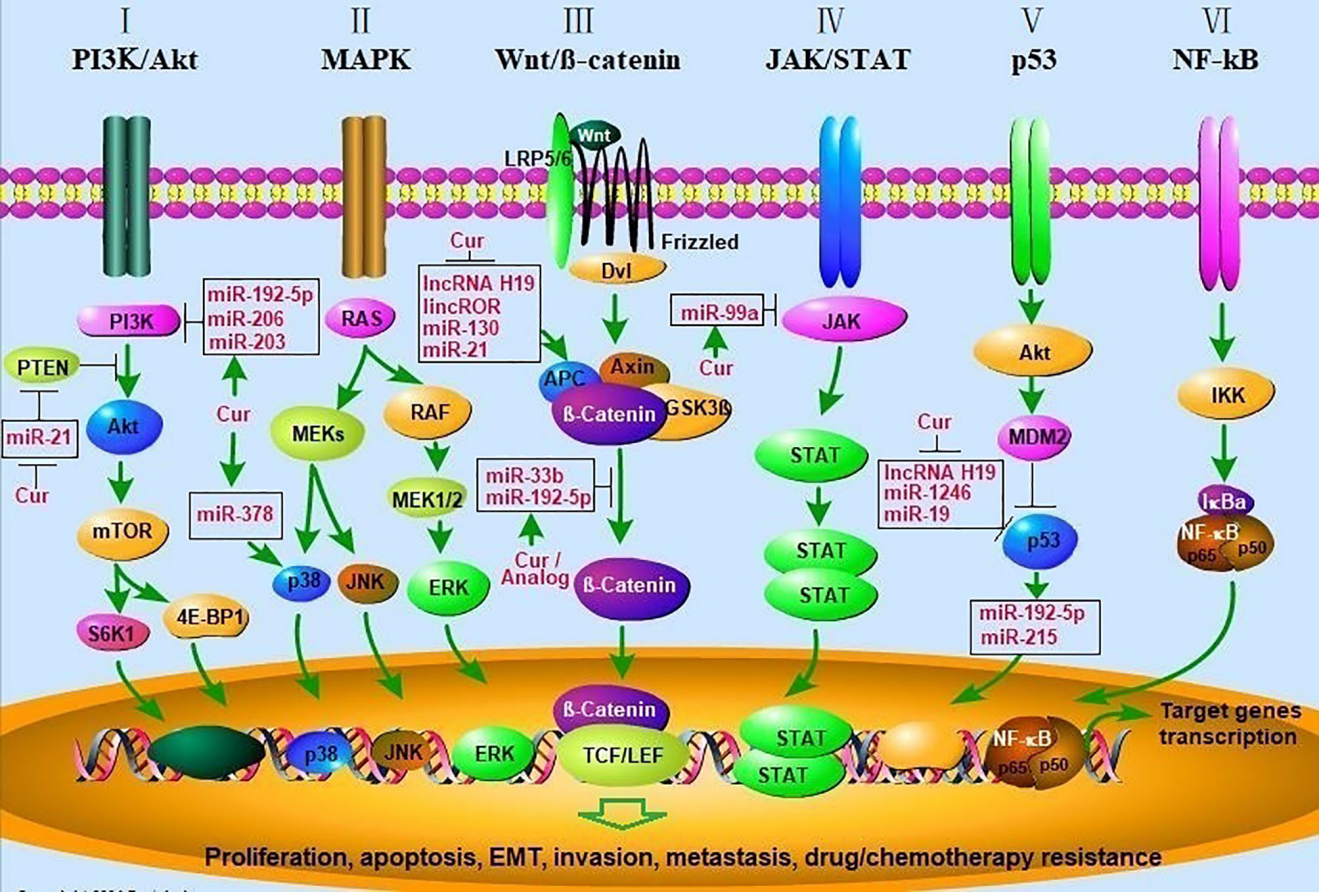
Curcumin modulates cancer progression by regulating multiple signal transduction pathways. (I) Akt/PI3K/mTOR signaling pathway. PTEN inhibits the activation of Akt by PI3K, mTOR phosphorylates p70S6K1 (S6K1) and 4E-BP1 leading to activation of pathways involved in cell growth and survival. Curcumin inhibit the Akt/PI3K/mTOR pathway by enhancing the activity of PTEN *via* decreasing miR-21 and inhibiting the activity of PI3K *via* upregulating miR-192-5p, miR-206, miR-203; (II) MAPK signaling pathway. Signaling cascades leading to activation of the MEKs, which lead to activation of the ERK1/2, p38, and JNK cascades, and then initiate the gene transcription. Curcumin activated p38 MAPK *via* upregulating miR-378, leading to elevated p21/27, cleaved-caspase-3,9 expression, decreased Bcl-2, MMP2/9 expression. (III) Wnt/β-catenin pathway. Wnt binds both Frizzled and LRP5/6 receptors to initiate the dissociation of the Axin/APC/GSK3β complex. β-catenin phosphorylation and then translocates to the nucleus to bind TCF/LEF co-transcription factors, which induces the Wnt-response gene transcription. Curcumin inhibits the Wnt/β-catenin pathway by inhibiting lncRNA H19, lincROR, miR-130, 21 and upregulating miR-192-5p and miR-33b; (IV) JAK/STAT signaling. The pathway is activated by the binding of a ligand and then convey signals downstream STATs, whereas STATs are transcription factors that activate gene expression; (V) p53 signaling pathway. AKT-induced activation of MDM2, which can inhibit the antitumor activity of p53. Curcumin enhances the anti-tumor activity of p53 by inhibiting lncRNA H19, miR-1246, miR-19; (VI) NF-kB signaling pathway. Signaling cascade leads to the phosphorylation of IkBα resulting in degradation by the proteasome. This releases the NF-kB/p65/p50 complex and allows it to translocate to the nucleus for gene transcription. AKT, v-akt murine thymoma viral oncogene; PI3K, phosphatidylinositol 3-kinase; mTOR, mammalian target of rapamycin; PTEN, phosphatase and tensin homolog; MAPK, mitogen-activated protein kinase; MMP, matrix metalloprotinase), ERKs (extracellular regulated protein kinases), JNK (c-Jun N-terminal kinase; APC, adenomatous polyposis coli; Dvl, Disheveled; GSK3β, glycogen synthase kinase 3β; LRP5/6, low density lipoprotein receptor-related protein 5/6; TCF/LEF, T-cell factor/lymphoid enhancer-binding factor; JAK/STAT, janus kinase/signal transducers and activators of transcription; MDM2, murine double minute 2; NF-κB, nuclear factor kappa B; IKK, IκB kinase; IκBa, IkappaBa.

Natural compounds have been well known for their potential effect on preventing cancer progression and as complementary or stand-alone therapies for cancer treatment (19, 118, 119). As a natural phenolic compound, curcumin is extracted from the dietary spice turmeric, showing an important significance in the adjuvant treatment of tumors with non-toxicity and tolerability (120–123). Curcumin participates in tumor control through multiple signaling pathways, including PI3K/Akt, JAK/STAT, MAPK, Wnt/β-catenin, p53, NF-ĸB and apoptosis related signaling pathways (**Table 1**). In addition, it is well known that ncRNA (including miRNA and lncRNA) plays an important role in the development of tumors. Curcumin regulates the expression of ncRNAs, which in turn affects the expression of related signaling pathway genes/proteins, and ultimately inhibits tumor cell proliferation, promotes cell apoptosis, and enhances sensitivity to chemotherapy drugs (**Figure 1**). These findings will help us better understand the molecular mechanism between curcumin and ncRNAs in anti-tumor, and the potentiality of clinical adjuvant treatment. Collectively, curcumin is a natural compound with potential drug value in adjuvant treatment of tumors.

**Table 1:** Curcumin modulates cellular signaling pathway and ncRNAs in cancers.

Curcumin or analog	Signal Pathways	ncRNAs*	Downstream Targets	Cancer Type	Refs
Curcumin	PI3K/Akt	miR-206 (↑) miR-192-5p (↑) miR-203 (↑) miR-21 (↓)	p-Akt (↓), p-mTOR (↓); Caspase-3 (↑), PI3K (↓), Akt (↓); Akt2 (↓), Src (↓), Jun (↓), Survivin (↓); PTEN (↑), PI3K (↓), Akt (↓), p-Akt (↓); p21 (↑), MMP2 (↓), MMP9 (↓);	Non-small cell lung cancer Non-small cell lung cancer Bladder cancer Gastric cancer Gastric cancer	(23) (24) (30) (27, 28)
Curcumin	JAK-STAT	miR-99a (↑)	p-JAK1 (↓), p-STAT1, 3 (↓), CyclinD1 (↓), p21 (↑), Bcl-2 (↓), Bax (↑), Cleaved-caspase-3,9 (↑), MMP2 (↓), RhoA (↓), ROCK1 (↓), Vimentin (↓);	Retinoblastoma	(37)
Analog	Wnt/β-catenin	miR-33b (↑)	HMGA2 (↓), E-cadherin (↑), N-cadherin (↓), Vimentin (↓), p-STAT3 (↓);	Melanoma	(53)
Curcumin		miR-192-5p (↑)	Cyclin D1 (↓), c-Myc (↓), β-catenin (↓);	Non-small cell lung cancer	(45)
		miR-21 (↓)	Bcl-2 (↓), Bax (↑), RECK (↑), MMP2 (↓), c-Myc (↓), β-catenin (↓), GSK-3β (↓);	Osteosarcoma	(44)
		miR-130 (↓)	Nkd2(↓), β-catenin (↓), TCF4 (↓);	Colon cancer	(46)
		lincROR (↓)	β-catenin (↓), CD44 (↓), Oct3/4 (↓), CyclinD1 (↓), c-Myc (↓);	Hepatocellular carcinoma	(51)
		lncRNA H19 (↓)	E-cadherin (↑), N-cadherin (↓), Vimentin (↓);	Breast cancer	(57)
Curcumin	MAPK	miR-378 (↑)	p-p38 (↑);	Glioblastoma	(62)
		/	p-JNK (↑), Cyclin D3 (↓), CDK2,6 (↓), p21 (↑), p27 (↑), Cleaved-caspase-3,9 (↑), p-p38 (↑);	Retinoblastoma	(63)
		/	p-JNK (↑), p-p38 (↑), p-ERK1,2 (↓), p-p65 (↓), MMP2 (↓), MMP9 (↓), Vimentin (↓);	Leukaemia	(64)
		/	p-p38 (↑), p-JNK (↑), Cleaved-caspase-3,9 (↑), Bcl-2 (↓), Bax (↑), HSP70 (↓); Cleaved-PARP (↑), ATF2 (↑), NF-κB (↓);	Non-small cell lung cancer Colon cancer	(65) (66)
Curcumin	p53	miR-19 (↓)	p53 (↑), p-MDM2 (↓), PCNA (↓), p-Akt (↓), PTEN (↑);	Breast cancer	(74)
		miR-1246 (↓)	p53 (↑);	Bladder cancer	(78)
		lncRNA H19 (↓)	p53 (↑), Bcl-2 (↓), Bax (↑), c-Myc (↓);	Gastric cancer	(79)
		miR-192-5p (↑) miR-215 (↑)	p53 (↑), p21 (↑), Cleaved-PARP (↑), Cleaved-caspase-3 (↑), XIAP (↑);	Non-small cell lung cancer	(75)
Curcumin	NF-ĸB	/	p-ERK (↓), p-NF-ĸB (↓), MMP9 (↓), Vimentin (↓); Fas (↑), FADD (↑), Cleaved-caspase‑3,8 (↑), MMP‑9 (↓), NF‑κB (↓), E-cadherin (↑), claudin‑3 (↓),	Pancreatic cancer Colon cancer	(85) (86)

*ncRNAs expression altered under curcumin treatment.

## Author Contributions

All authors listed have made a substantial, direct, and intellectual contribution to the work, and approved it for publication.

## Funding

This work was supported by National Natural Science Foundation of China (U1904133,81903688), Program for Young Key Teachers in Colleges and Universities in Henan Province (2020GGJS150), the Natural Science Foundation of Henan Province of China (212300410224), and Scientific Research Foundation of Xinxiang Medical University (XYBSKYZZ201512). This work was also supported by National Training Programs of Innovation and Entrepreneurship for Undergraduates (202010472011).

## Conflict of Interest

The authors declare that the research was conducted in the absence of any commercial or financial relationships that could be construed as a potential conflict of interest.
